# Risk Stratification of Operatively Treated Intertrochanteric Hip Fractures Reveals Differences in Short-Term Outcomes and Procedure Costs Between Sliding Hip Screw Versus Short Cephalomedullary Nail

**DOI:** 10.5435/JAAOSGlobal-D-21-00242

**Published:** 2021-12-07

**Authors:** Sanjit Konda, Rachel Ranson, Ariana Lott, Tensae Assefa, Joseph Johnson, Abhishek Ganta, Kenneth Egol

**Affiliations:** From the Division of Orthopedic Trauma Surgery, Department of Orthopedic Surgery, NYU Langone Health, NYU Langone Orthopedic Hospital, New York (Dr. Konda, Dr. Ranson, Dr. Lott, Assefa, Johnson, Dr. Ganta, Dr. Egol); and the Department of Orthopedic Surgery, Jamaica Hospital Medical Center, Queens (Dr. Konda, Dr. Ganta), NY.

## Abstract

This study sought to retrospectively compare short-term outcomes between sliding hip screw (SHS) fixation versus short cephalomedullary nailing (CMN) in a risk-stratified cohort of geriatric intertrochanteric hip fracture patients. Data of 458 patients, aged 55 years and older, who sustained intertrochanteric fractures (OTA 31A1-3) and were treated with either SHS or short CMN from October 2014 to March 2019 were collected. Patient demographics, injury severity, and functional status were used to calculate a score using the Score for Trauma Triage in the Geriatric and Middle-Aged system. Based on the Score for Trauma Triage in the Geriatric and Middle-Aged tool, 229 patients (50%) were placed into a low-risk cohort, whereas 229 patients (50%) were placed into a high-risk cohort.

**Results:**

Four hundred eleven patients were treated with CMN and 47 patients were treated with SHS. Procedural time was shorter for low- and high-risk patients treated with CMN versus SHS. Need for transfusion did not differ between implant types in either risk cohort. In the high-risk cohort, incidence of major complications and number of overall inpatient complications were higher in the SHS group. In the low-risk cohort, patients with SHS were discharged home more often and ambulated a greater distance before discharge. Although total costs did not differ between groups, procedural costs were lower in the SHS group for both risk cohorts. Multivariate analyses demonstrated that implant type was a significant predictor of all aforementioned significant bivariate analyses.

**Conclusion:**

In low-risk and high-risk patients, those treated with CMN had shorter surgical time but higher procedural costs. A decrease in implant cost may optimize the value of shorter procedural times associated with CMN use, especially for high-risk patients. Our results suggested that high-risk hip fracture patients should be treated with CMN for both stable and unstable fractures and low-risk stable fracture patterns should be treated with SHS.

Intertrochanteric fractures in the elderly population are commonly encountered and are expected to increase in frequency over the next several decades.^1^ As a result, these fractures are projected to create an economic strain on the US healthcare system with a doubling of the annual hip fracture cost to $16 billion by 2040.^2^ Treatment modalities have evolved over time, with recent trends indicating an increase use in cephalomedullary nailing (CMN) devices over extramedullary devices, such as sliding hip screws (SHS), despite an increase in implant cost.^3^ Multiple studies comparing extramedullary and intramedullary implants have demonstrated superior biomechanical properties and anatomical results with intramedullary implants. However, large-scale studies have not demonstrated any notable difference in clinical outcomes for patients treated with either implant.^4,5,6,7^ A recent National Surgical Quality Improvement Program database study demonstrated a shorter length of stay for patients treated with intramedullary fixation. However, the study did not delve into patient risk factors or fracture morphology.^8^

The purpose of this study was to determine whether risk stratification can differentiate patient outcomes at varying levels of risk based on implant type. We hypothesize that the use of risk stratification can identify different outcomes based on implant choice for fixation of intertrochanteric fractures in the geriatric population. To our knowledge, no study has attempted to compare intramedullary versus extramedullary implant use in a risk-stratified cohort of geriatric hip fracture patients.

## Methods

After Institutional Review Board approval, all patients aged 55 years and older with low-energy intertrochanteric hip fractures (OTA 31A1-3) admitted to a level 1 trauma center and one orthopedic specialty hospital between October 2014 and March 2019 were identified. Patients who were treated operatively using either a SHS construct or a short cephalomedullary nail construct met initial the inclusion criteria. All high-energy mechanism of injury fractures including motor vehicle accidents, motorcycle accidents, fall from height (greater than two stairs), and pedestrian struck injuries were excluded. Low-energy mechanisms were defined as mechanical falls or falls from fewer than two stairs.

On initial presentation to the emergency department, each patient's demographics, injury severity, preexisting co-morbidities, and functional status were used to calculate a score for trauma triage in the geriatric and middle-aged (STTGMA)—a risk stratification scoring system that was previously validated using the National Trauma Databank.^9,10^ STTGMA variables are reported in Table 1. The STTGMA system calculates a predicted inpatient morality risk on a scale of 0% to 100%. Based on the STTGMA system, patients were divided into two risk cohorts: low-risk (range: 0% to 0.39%) and high-risk (range: 0.4% to 22.5%) groups. The cut point was determined by separating the lowest 50% of STTGMA from the highest 50% STTGMA. This STTGMA risk stratification scheme is similar to those used in previous studies to adequately stratify patients from low to high risk.^10,11,12,13^

**Table 1:** Demographics and STTGMA Variables for all 458 Patients Across Implant Type

Characteristic	Sliding Hip Screw (N = 47)	Short Cephalomedullary Nail (N = 411)	*P*
Age^a^	78.5 ± 11.5	83.6 ± 9.5	**<0.001**
Female sex	27 (57%)	297 (72%)	**0.042**
Glasgow Coma Scale^a^	14.9 ± 0.4	14.8 ± 0.8	0.496
Abbreviated injury severity—head^a^	0.04 ± 0.30	0.04 ± 0.20	0.979
Abbreviated injury severity—chest^a^	0.04 ± 0.20	0.01 ± 0.13	0.157
Charlson comorbidity index^a^	1.3 ± 1.5	1.4 ± 1.5	0.669
Ambulatory status^a^ ^b^	1.3 ± 0.5	1.3 ± 0.5	0.615
STTGMA (%)	1.2 ± 2.9	1.2 ± 2.6	0.962

STTGMA = score for trauma triage in the geriatric and middle-aged.

a Denotes STTGMA variables.

b Ambulatory status is graded on a 3-point scale: 1 = community ambulator, 2 = household ambulator, and 3 = wheelchair or bed-bound.

Bolded entries are for *P* values <0.05 indicating statistical significance.

Plain radiograph films and occasional CT imaging were reviewed for fracture classification. Three fellowship-trained traumatologists conducted fracture classification. The short CMN was used for unstable fractures (OTA 31A2.1, 31A2.2, and 31A3), whereas both the SHS and short CMN were used for stable fractures (OTA 31A1.2 and 31A1.3). The decision to treat stable fracture patterns with SHS versus CMN was based on surgeon preference. Fractures of the greater trochanter were excluded from analysis (OTA 31A1.1). All surgery was done by resident trainees' staff under the supervision of an attending orthopedic trauma surgeon. Fracture type, length of stay, need for advanced level of care including intensive care and step down care, inpatient complications, inpatient mortality, need for transfusion as per the institutional transfusion protocol criteria, and ambulation distance at day of discharge were recorded.

Minor complications included urinary tract infection, acute anemia, acute kidney injury, surgical site infection, and decubitus ulcer, and major complications included septic shock, pneumonia, acute respiratory failure, acute myocardial infarction, deep vein thrombus, pulmonary embolism, cardiac arrest, and stroke. Procedure details including implant choice, anesthesia type, and surgical time (time from incision to closure) were also recorded. Cost data were obtained from the hospital finance department at one of the hospitals (level 1 trauma center) for 45% of the patients included in this study with an equal distribution of low-risk and high-risk patients. Cost data were then extrapolated to rest of the patients. Cost was divided into the following subcategories: room/board, emergency department, pharmacy, laboratory/pathology, radiology, dialysis, cardiology, procedure, allied health, and other (eg, blood).

Patients were placed into low-risk and high-risk cohorts, and those treated with CMN were compared with those treated with SHS using both bivariate and multivariate analyses. Bivariate comparisons used independent samples *t*-test, chi-square test, and Fisher exact test, as appropriate. Multivariate linear and logistic regression analyses used forward variable selection with threshold for entry set at *P* < 0.05 for demographic, injury, and functional status differences between patients with SHS and CMN after risk-group stratification. Statistical analyses were done using IBM SPSS software version 25 (IBM Corporation). Statistical significance was set a priori at *P* < 0.05.

## Results

This study included 458 patients with intertrochanteric fractures treated operatively with either an SHS or a CMN construct. Of these, 145 (32%) were 31A1.2 and 31A1.3 fractures, 279 (61%) were 31A2.1 and 31A2.2 fractures, and 34 (7%) were 31A3 fractures. OTA 31A1.2 and 31A1.3 fractures (32%) were considered stable, whereas 31A2.1, 31A2.2, and 31A3 fracture patterns (68%) were unstable. Four hundred eleven patients (89.7%) were treated with CMN and 47 patients (10.3%) with SHS.

Although mean age differed between the CMN and SHS groups (78.5 versus 83.6, *P* = 0.001), STTGMA did not vary (1.2% versus 1.2%, *P* = 0.962, respectively). No other differences were observed in variables incorporated into the STTGMA algorithm between cohorts (Table 1). Although female sex was more prevalent in the short CMN cohort (*P* = 0.042), sex is not a variable included in the STTGMA system.

Two hundred twenty-nine patients (50%) were grouped into the low-risk cohort with a mean STTGMA of 0.2% ± 0.1%, and 229 patients (50%) were included in the high risk cohort with a mean STTGMA of 2.2% ± 3.4%. Demographic breakdowns of the CMN and SHS groups by risk-stratified cohorts are detailed in Tables 2 and 3. When stable and unstable fracture patterns were compared by STTGMA system, univariate analysis revealed no difference. The mean STTGMA for stable and unstable fracture patterns was similar (0.012 + 0.026 for both; *P* = 0.938), demonstrating that fracture patterns were not associated with a patients initial mortality risk assessment.

**Table 2:** Demographic and STTGMA Variables for the High-Risk Cohort Across Implant Type

Characteristic	Sliding Hip Screw (N = 16)	Short Cephalomedullary Nail (N = 213)	*P*
Age	87.6 ± 7.1	86.5 ± 7.8	0.571
Female sex	9 (56.3%)	146 (68.5%)	0.406
Glascow coma scale	14.75 ± 0.6	14.69 ± 1.0	0.816
Abbreviated injury severity—head	0.13 ± 0.3	0.08 ± 0.4	0.668
Abbreviated injury severity—chest^a^	0.13 ± 0.3	0.01 ± 0.1	**0.003**
Charlson comorbidity index	2.1 ± 2.2	2.2 ± 1.5	0.879
Ambulatory status	1.9 ± 0.3	1.6 ± 0.6	0.115
STTGMA (%)	3.1 ± 4.4	2.1 ± 3.3	0.263

STTGMA = score for trauma triage in the geriatric and middle-aged.

a Variable controlled for in the multivariate analysis based on statistical significance.

Bolded entries are for *P* values <0.05 indicating statistical significance.

**Table 3:** Demographic and STTGMA Variables for the Low-Risk Cohort Across Implant Type

Characteristic	Sliding Hip Screw (N = 31)	Short Cephalomedullary Nail (N = 198)	*P*
Age^a^	73.7 ± 10.5	80.6 ± 10.2	**0.001**
Female sex^a^	18 (58.1%)	151 (76.3%)	**0.046**
Glascow coma scale	14.97 ± 0.2	14.95 ± 0.2	0.739
Abbreviated injury severity—head	0	0	—
Abbreviated injury severity—chest	0	0.01 ± 0.1	0.693
Charlson comorbidity index^a^	0.8 ± 0.8	0.5 ± 0.6	**0.005**
Ambulatory status	1.0 ± 0.1	1.0 ± 0	0.576
STTGMA (%)	0.2 ± 0.1	0.2 ± 0.1	0.598

STTGMA = score for trauma triage in the geriatric and middle-aged.

a Variable controlled for in the multivariate analysis based on statistical significance.

Bolded entries are for *P* values <0.05 indicating statistical significance.

No difference in length of stay, need for intensive care unit or step down unit level care, or minor complication rates was found between the patients implanted with CMN and SHS in both the high-risk and low-risk cohorts. High-risk patients with SHS implants experienced more major complications (such as septic shock, pneumonia, acute respiratory failure, acute myocardial infarction, deep vein thrombus, pulmonary embolism, cardiac arrest, and stroke) and a greater number of inpatient complications (both major and minor complications) than the CMN group (Table 4).

**Table 4:** Bivariate Analysis for Hospital Quality Measures and Short-Term Outcomes for the High-Risk Cohort (STTGMA Range: 0.4% to 22.5%)

	Sliding Hip Screw (N = 16)	Short Cephalomedullary Nail (N = 213)	*P*
Inpatient mortality	2 (12.5%)	6 (2.8%)	0.100
1-yr mortality	3 (18.8%)	27 (12.7%)	0.350
Major complications	6 (37.5%)	28 (13.1%)	**0.018**
Minor complications	12 (75%)	129 (60.6%)	0.298
Total no. of complications	2.3 ± 1.4	1.1 ± 1.1	**<0.001**
Need for blood transfusion	11 (68.8%)	115 (54%)	0.304
Intensive care unit stay	6 (37.5%)	43 (20.2%)	0.117
Discharged home	0	24 (11.3%)	0.387
Spinal anesthesia	6 (37.5%)	51 (23.9%)	0.237
Ambulatory distance before discharge (feet)	7.3 ± 9.4	10.3 ± 21.2	0.572
Average postoperative day evaluated for ambulation	5.8 ± 3.6	5.4 ± 3.8	0.634
Length of stay (days)	7.9 ± 3.8	7.5 ± 4.1	0.653
Procedural time (min)	73.4 ± 12.5	51.8 ± 18.1	**<0.001**
Total cost (US dollar)	24,958 ± 8152	25,693 ± 7973	0.787
Procedural cost (US dollar)	6590 ± 653	9124 ± 1400	**<0.001**

Bolded entries are for *P* values <0.05 indicating statistical significance.

Conversely, low-risk patients treated with SHS had the same inpatient mortality rate (*P* = 1.000) as those with CMN and were discharged home more frequently (*P* = 0.004). The low-risk SHS cohort ambulated greater distances before discharge than those with CMN (*P* < 0.001). However, these patients were also evaluated by physical therapy an average of a day and a half later than those with CMN (*P* = 0.049) (Table 5). Neither inpatient nor one-year mortality rates differed in both high-risk and low-risk cohorts across implant type (Tables 4 and 5).

**Table 5:** Bivariate Analysis for Hospital Quality Measures and Short-Term Outcomes for the Low-Risk Cohort (STTGMA Range: 0% to 0.39%)

	Sliding Hip Screw (N = 31)	Short Cephalomedullary Nail (N = 198)	*P*
Inpatient mortality	0%	2 (1%)	1.000
1-yr mortality	0%	5 (2.5%)	0.480
Major complications	1 (3.2%)	19 (9.6%)	0.489
Minor complications	14 (45.2%)	107 (54%)	0.440
Total number of complications	1.2 ± 0.2	1.1 ± 0.08	0.947
Need for blood transfusion	8 (25.8%)	87 (43.9%)	0.077
Intensive care unit stay	3 (9.7%)	12 (6.1%)	0.435
Discharged home	15 (48.4%)	45 (22.7%)	**0.004**
Spinal anesthesia	8 (25.8%)	53 (26.9%)	1.000
Ambulatory distance Before discharge (feet)	68.0 ± 72.4	27.2 ± 39.6	**<0.001**
Average postoperative day evaluated for ambulation	6.2 ± 7.3	4.7 ± 3.1	**0.049**
Length of stay (d)	7.8 ± 7.5	6.5 ± 3.5	0.101
Procedural time (min)	69.9 ± 19.5	52.7 ± 21.7	**<0.001**
Total cost (US dollar)	23,650 ± 12,689	23,895 ± 8534	0.931
Procedural cost (US dollar)	7189 ± 1211	9402 ± 2161	**0.001**

Bolded entries are for *P* values <0.05 indicating statistical significance.

Procedural time was significantly shorter (17 minutes and 22 minutes, respectively) for low-risk and high-risk cohorts using a CMN vs SHS (*P* < 0.001 and *P* < 0.001, respectively). However, procedural costs were $2213 and $2534 higher for CMN versus SHS in the low-risk and high-risk cohorts (*P* = 0.001 and *P* < 0.001), respectively. No difference was found in total cost of hospitalization between patients implanted with SHS and CMN for either risk-stratified group (Tables 4 and 5).

After adjusting for relevant demographic, injury, and functional status covariates, multivariate analysis demonstrated that high-risk patients who were treated with an SHS implant experienced significantly longer procedural times relative to those treated with a CMN (B coefficient: 20.956, 95% confidence interval [CI]: 11.708 to 30.204, *P* < 0.001) (Table 6). In addition, high-risk patients treated with SHS experienced a higher number of total complications (B coefficient: 1.097, 95% CI: 0.491 to 1.703, *P* < 0.001) and higher odds of developing a major complication (odds ratio [OR]: 3.557, 95% CI: 1.160 to 10.900, *P* = 0.026) relative to CMN patients. Similarly, low-risk patients treated with SHS witnessed longer procedural times relative to those treated with CMN (B coefficient: 15.317, 95% CI: 6.779 to 23.855, *P* < 0.001) (Table 7). Among low-risk patients, treatment with SHS was also associated with greater ambulatory distance before discharge (B coefficient: 31.504, 95% CI: 14.784 to 48.225, *P* < 0.001) and higher odds of home discharge after the index hospitalization (odds ratio [OR]: 2.398, 95% CI: 1.023 to 5.621, *P* = 0.044).

**Table 6:** Multivariate Analyses for High-Risk Patients Treated With Sliding Hip Screw versus Short Cephalomedullary Nails (Reference Group)

Quality Measure	Variables	B Coefficient	95% CI	*P*
Procedural time (min)	Implant type	20.956	11.708 to 30.204	<0.001
	AIS—chest^a^	6.556	−9.574 to 22.685	0.424
No. of complications	Implant type	1.097	0.491 to 1.703	<0.001
	AIS—chest^a^	0.238	−0.819 to 1.295	0.658

Quality Measure	Variables	Odds Ratio	95% CI	*P*
Major complication (number)	Implant type	3.557	1.160-10.900	0.026
	AIS—chest^a^	0.962	0.373-18.362	0.333

a Abbreviated injury severity score—chest.

**Table 7:** Multivariate Analyses for Low-Risk Patients Treated With Sliding Hip Screw versus Short Cephalomedullary Nails (Reference Group)

Quality Measure	Variables	B Coefficient	95% CI	*P*
Procedural time (min)	Implant type	15.317	6.779 to 23.855	<0.001
	Age	−0.103	−0.383 to 0.176	0.466
	Sex	−3.588	−10.054 to 2.878	0.275
	CCI^a^	1.421	−3.010 to 5.851	0.528
Ambulatory distance before discharge (feet)	Implant type	31.504	14.784 to 48.225	<0.001
	Age	−1.596	−2.142 to −1.050	<0.001
	Sex	−9.766	−22.422 to 2.890	0.130
	CCI^a^	−9.54	−18.2016 to −0.875	0.031

Quality Measure	Variables	Odds Ratio	95% CI	*P*
Discharged home	Implant type	2.398	1.023-5.621	0.044
	Age	0.935	0.906-0.966	<0.001
	Sex	0.724	0.361-1.453	0.363
	CCI^a^	0.742	0.458-1.201	0.224

a Charlson comorbidity index.

## Discussion

The use of the STTGMA tool allows for inpatient mortality risk stratification that was validated within the National Trauma Databank.^9^ Risk stratification separates patients into high-risk and low-risk cohorts, thus controlling for the confounding factor of patient characteristics and comorbidities. Although outcomes for patients with intertrochanteric fractures treated with SHS or CMN were analyzed in prior studies, to our knowledge, none of these studies risk-stratified patients using a validated tool.

Osteoporotic fractures such as intertrochanteric fractures pose a difficult challenge for orthopedic surgeons. An increasing trend in the use of CMN devices over extramedullary devices for treatment of intertrochanteric fractures, most notably in unstable fractures, is noted.^3^ Biomechanically, the CMN is a more stable construct; however, the clinical implications for this have yet to be fully elucidated.^4,5,6,7^ The largest study to date showed that CMN were associated with an increased risk of peri-implant fracture; however, recent implant design modification and repeat analysis have showed no such risk.^14,15^ Furthermore, additional studies have noted no notable difference in mortality, postoperative pain, postoperative mobility, or in-hospital complications between the two implants.^7^

By subdividing patients into low-risk and high-risk groups, we are able to analyze more frail patients who are at an increased risk of complications separately from less frail patients. In our univariate analysis, risk-stratifying patients demonstrated that in low-risk patients, those implanted with SHS were more likely to be discharged home and were able to ambulate further before discharge. However, the longer ambulatory distance could be confounded by greater delay in physical therapy evaluation of SHS compared with patients implanted with CMN. In the high-risk cohort, several notable differences were observed in patient outcome favoring CMN use, particularly regarding decreased procedural time, decreased major complications, and decreased number of inpatient complications. Thus, risk stratification of patients suggests that low-risk patients benefit from SHS and high-risk patients benefit from short CMN use.

Because decreased procedural time with CMN use was consistent among low-risk and high-risk cohorts, a multivariate analysis was done to determine contributing factors; only implant choice was shown to effect procedural time. Because of the high volume of IT fixation with short CMN at this institution, surgeon and staff familiarity with this procedure may play a role in shorter procedural time; however, this finding was noted in other studies as well.^16,17^ Shorter procedural time was not found to contribute to mortality, complications, transfusion need, or length of stay; therefore, we believe that shorter procedural time does not affect short-term patient outcomes as measured in this study. There are, however, benefits to shorter procedural time: A decrease in operating room time per case can generate an increase in total surgical cases per day or can allow for longer cases to be completed within standard shifts to avoid overtime.^18,19^

For stable fractures, an SHS provides value in lower implant cost and earlier discharge from the hospital, but for the higher risk patients, that benefit is diminished. In addition, the results of this article suggest that for high-risk patients (>0.4% mortality risk) identified by the STTGMA tool, using a CMN in all stable and unstable intertrochanteric fractures patterns (OTA 31A1-3) may have a value advantage. In this cohort of patients, CMN use is associated with a lower major complication rate and overall number of inpatient complications. However, CMN use is associated with a higher procedure cost despite decreased procedural time primarily because of the higher cost of the CMN implant. However, the scope of this article was not to determine the level of increased cost that is considered acceptable to achieve the level of improved outcomes observed in this study. However, our stance is that overall improved value is achieved with the CMN in high-risk patients based on our study results and that even further value advantage can be achieved by reduction of CMN implant cost.

This study has several limitations. The retrospective nature of this study is subject to errors of confounding and bias due to the reliance on accurate record keeping. Because this study evaluated only short-term outcomes, we could not take into consideration factors such as fixation failure and revision surgery rate, both of which would affect a value analysis. Finally, the cost analysis provided here is limited to a single institution and will vary on a hospital-to-hospital basis, which in turn may affect the value analysis.

## Conclusion

Risk stratification analysis of implant choice for treatment of intertrochanteric hip fracture seems to alter the traditional value analysis between SHS and CMN. Patients in both high-risk and low-risk cohorts treated with CMN had shorter surgical time but higher procedural cost. A value-based treatment algorithm resulting from this study is that low-risk stable intertrochanteric hip fractures should receive an SHS because of improved outcome measures. High-risk stable and unstable intertrochanteric hip fractures should receive CMN because of improved inpatient complication measures. Additional value with CMN use could potentially be obtained in both the low-risk and high-risk cohorts if implant costs are decreased. This study demonstrated how the STTGMA risk stratification method can be used to determine implant value in a risk-stratified cohort of fracture patients.
